# Vascularized bone grafts to the upper extremities

**DOI:** 10.1186/1753-6561-9-S3-A57

**Published:** 2015-05-19

**Authors:** Keiichi Murata

**Affiliations:** 1 Limb Trauma Center, Nara City Hospital, Nara, 630-8305, Japan

## Introduction

Vascularized bone grafts have been used in patients with various kinds of intractable diseases. From 1979 to 2013, we performed 89 vascularized bone grafting for reconstruction of upper extremities. We reviewed results and postoperative complications of these cases.

## Materials and methods

The reconstructed site was the humerus in 15 cases, the radius, the ulna or the wrist joint in 22 cases, carpal or metacarpal bone in 52. The etiologies consisted of three traumatic bone defects, 13 traumatic nonunions of long bone, two osteonecroses of the humeral head, eight osteomyelitis, nine case after resections of bone tumor, two congenital pseudoarthroses of the ulna, one congenital club hand, 23 nonunions of the scaphoid fracture, 26 Kienböck’s deseases and two Preiser’s desease. As the donor site, we used fibula graft for segmental bone defects by trauma or after resection of bone tumor, pedicled scapula graft for lesions around the head or neck of humerus, thin corticoperiosteal graft from the medial femoral condyle for nonunions with small bone defect, vascularlized bone from the distal radius for nonunions of scaphoid fractures, Kienböck’s diseases or Preiser’s diseases.

## Results

Postoperative circulatory disturbance necessitated revision surgery in three cases (venous thrombosis in two, arterial and venous thrombosis in one). Malunion occurred in four cases. Gradual varus deformity of the forearm was developed in two. Additional bone grafting was needed in one case. Bone union was not achieved in five cases (bone union rate 96%).

## Conclusion

Vascularized fibula graft is good indication for the patients with large bone defects in the upper extremities. Pedicled scapular graft is useful for lesions around the head or neck of humerus. For nonunions with small bone defects thin corticoperiosteal graft is preferable with less invasive procedure. Vascularized bone graft from the distal radius (e.g. 1.2 ICSRA, 4.5ICA etc.) is useful for nonunions of scaphoid fractures or Kienböcks diseases (Stage II or III).

